# Introductory Lectures

**Published:** 1855-10

**Authors:** 


### INTRODUCTORY LECTURES.

The season for Introductory Lectures has returned, and these pleasing annuals are pouring in upon us. We hope to find leisure to remark upon the merits of some of them during the winter. Among those which lie before us we cannot help speaking of the excellence of Prof. Dalton's, delivered in the New York College of Physicians and Surgeons.

